# Exploring the effects of COVID-19 outbreak control policies on services offered to people experiencing homelessness

**DOI:** 10.1186/s12889-024-20312-3

**Published:** 2024-10-14

**Authors:** Alexa J. Davis, Donna M. Halperin, Brian R. Condran, Melissa S. Kervin, Antonia M. Di Castri, Katherine L. Salter, Julie A. Bettinger, Janet A. Parsons, Scott A. Halperin

**Affiliations:** 1 Rankin School of Nursing, St. Francis Xavier University, 4130 University Ave, Antigonish, NS B2G 2W5 Canada; 2 Canadian Center for Vaccinology, Dalhousie University, IWK Health, Nova Scotia Health, Goldbloom RCC Pavilion, 4th floor, 5850/5980 University Avenue, Halifax, NS B3K 6R8 Canada; 3 Nova Scotia Health Learning Institute for Healthcare Providers, Nova Scotia Health, Halifax, NS Canada; 4 Departments of Pediatrics and Microbiology & Immunology, Dalhousie University, 5850/5980 University Ave, Halifax, NS Canada; 5 Vaccine Evaluation Center, BC Children’s Hospital Research Institute, Vancouver, BC Canada; 6 Department of Pediatrics, University of British Columbia, Vancouver, BC Canada; 7 Department of Occupational Science and Occupational Therapy, Temerty Faculty of Medicine, University of Toronto, Toronto, ON Canada; 8 Li Ka Shing Knowledge Institute, St. Michael’s Hospital, Unity Health Toronto, Toronto, ON M5B 1W8 Canada

**Keywords:** Non-profit organizations, Public Health Outbreak Control Policies, Rural services, Qualitative research, Pandemics, Canada

## Abstract

**Background:**

The COVID-19 pandemic and subsequent implementation of public health policies exacerbated multiple intersecting systemic inequities, including homelessness. Housing is a key social determinant of health that played a significant part in the front-line defence against COVID-19, posing challenges for service providers working with people experiencing homelessness (PEH). Public health practitioners and not-for-profit organizations (NFPs) had to adapt existing COVID-19 policies and implement novel measures to prevent the spread of disease within congregate settings, including shelters. It is essential to share the perspectives of service providers working with PEH and their experiences implementing policies to prepare for future public health emergencies and prevent service disruptions.

**Methods:**

In this qualitative case study, we explored how service providers in the non-profit sector interpreted, conceptualized, and implemented COVID-19 public health outbreak control policies in Nova Scotia. We interviewed 11 service providers between September and December 2020. Using thematic analysis, we identified patterns and generated themes. Local, provincial, and national policy documents were useful to situate our findings within the first year of the COVID-19 pandemic and contextualize participants’ experiences.

**Results:**

Implementing policies in the context of homelessness was difficult for service providers, leading to creative temporary solutions, including pop-up shelters, a dedicated housing isolation phone line, comfort stations, and harm reduction initiatives, among others. There were distinct rural challenges to navigating the pandemic, which stemmed from technology limitations, lack of public transportation, and service closures. This case study illustrates the importance of flexible and context-specific policies required to support PEH and mitigate the personal and professional impact on service providers amid a public health emergency. Innovative services and public health collaboration also exemplified the ability to enhance housing services beyond the pandemic.

**Conclusions:**

The results of this project may inform context-specific emergency preparedness and response plans for COVID-19, future public health emergencies, and ongoing housing crises.

**Supplementary Information:**

The online version contains supplementary material available at 10.1186/s12889-024-20312-3.

## Background

The coronavirus disease of 2019 (COVID-19) was declared a pandemic on March 11th, 2020, and has since had physical, mental, social, and economic impacts across the globe [1]. There is now substantial evidence on preventing infection and responding to COVID-19; however, not much was known about the novel virus in 2020. As vaccine development began, public health outbreak control policies were instrumental in containing and controlling the rapid spread of disease. Policies to keep people apart included isolation and quarantine requirements, physical distancing, travel restrictions, and working from home [2]. Other policies to reduce viral transmission included wearing personal protective equipment (such as masks), frequent testing, temperature checks, and rigorous cleaning measures [2]. While these public health policies helped to reduce the spread of COVID-19, they also exacerbated systemic inequities and adversely impacted the services offered to people experiencing homelessness (PEH) [3].

Homelessness is a systemic and societal issue, often caused by a lack of affordable and appropriate housing, discrimination, and financial, mental, behavioural, and physical challenges [4, 5]. PEH may include unsheltered, emergently sheltered, provisionally accommodated, or precariously housed individuals [4]. Homelessness is most visible in urban areas through encampments and people who sleep outside [6]. In comparison, rural homelessness is *“*hidden*”* as people often receive provisional housing from family, friends, or insecure spaces [4]. For individuals who can access emergency or provisional shelter, living conditions in a congregate setting include overcrowding and limited access to hygiene supplies. Lack of appropriate housing is also associated with chronic health conditions, placing people at an increased risk of contracting COVID-19, spreading it to others, and experiencing severe morbidity and mortality [7, 8]. PEH were considered a priority population for COVID-19 testing, contact tracing, and vaccination [9, 10]. Intersecting issues associated with homelessness, such as gender-based violence, poverty, food insecurity, substance use, and mental health conditions, increased the complexity of services required to adhere to these policies [11–13]. This study contains the perspectives of service providers who worked with PEH during the pandemic and navigated policy expectations in these settings. Due to technology limitations and distancing requirements, we did not include PEH in this study.

This study was conducted in Nova Scotia, a province on the east coast of Canada with a population of slightly over one million [14]. Most of the population lives in or around the capital city of Halifax, Nova Scotia’s largest urban municipality. Located in the north easternmost part of the province, Cape Breton-Sydney is the second most populous municipality. The rest of the province is relatively rural, with varying access to transportation, health services, and supportive housing [15]. We differentiated between urban and rural services to examine any differences in the pandemic response by place. Urban and rural settings were defined by population density and proximity to organizations that serve PEH in the community. Urban settings included Nova Scotia’s largest municipalities where most services for PEH are found, including the Halifax Regional Municipality, followed by the Cape Breton Regional Municipality [16, 17]. Rural settings included everywhere outside these locations. Our definition reflected the population and infrastructure available to support PEH in Nova Scotia.

According to the 2022 Point-in-Time count of people visibly experiencing homelessness or those connected to community-based supports, at least 586 people were without a safe and permanent address in Halifax on April 7th, 2022 [18]. Of note, the count parameters excluded people staying with friends and family, in hotels self-funded by employment or sex work, living in a home with gender-based violence, or living in rural areas of the province [18]. While these statistics are crucial for policymakers to allocate appropriate resources, the lack of rural data exacerbates funding disparities in addressing rural homelessness [19]. Most services for PEH in Nova Scotia operate within the non-profit sector, which consists of organizations mandated to address homelessness (emergency shelters, transition houses, food banks, street nurses and navigators, housing support programs, and other community-based organizations) and those whose mandate overlaps with homelessness (public health practitioners, social service agencies, substance use programs, mental health supports, library programming, and other health and social services). Organization and government policies, such as funding initiatives, rent caps, and subsidies, also impact these services [20].

At the beginning of the pandemic, limited context-specific guidance for PEH posed issues for service providers [21, 22]. These issues largely stemmed from a lack of pre-existing policies, programs, and infrastructure to support PEH, leading to gaps in COVID-19 policy implementation. In this context, a policy implementation gap is a difference between policy expectations and actual outcomes [23]. As a result, designated public health practitioners were assigned to assist not-for-profit organizations (NFPs) in implementing public health policies. In this paper, we discuss how service providers adapted their services to facilitate adherence with public health outbreak control policies during the COVID-19 pandemic.

## Methods

To guide our work in exploring the gaps in COVID-19 policy implementation, we asked the following research questions: (1) How do public health practitioners and NFPs that serve PEH in Nova Scotia interpret, conceptualize, and implement COVID-19 public health outbreak control policies? (2) What are the most critical factors supporting and inhibiting the implementation of COVID-19 public health outbreak control policies?

### Conceptual theories

Homelessness is a complicated social issue without one single cause or solution, and the services designed to support PEH operate within a dynamic system [24, 25]. The pandemic added another layer of complexity to these services, requiring collaboration between public health personnel, community-based organizations, transport services, volunteers, food services, healthcare providers, funding agencies, and many others to implement policies. As a human right and front-line defence against the pandemic, safe and stable housing is essential to protect and promote health and well-being [26]. Therefore, we used complexity theory to guide our understanding of the different components interacting within the non-profit sector [24].

Housing is a social determinant of health, a social condition in which people work and live that impacts overall health [26, 27]. The conceptual framework developed by Solar and Irwin [27] highlights the relationships between determinants of health and the impact of policies on these determinants. Housing includes both living conditions (such as weather, space, location, and quality) and social and public policy (such as labour market, social protection, public housing programs, and income supplements) [27]. Homelessness and inadequate housing policies can lead to health inequities, making it important to understand how these inequities impact different people, groups, and places [26]. Throughout this paper, we examine challenges during the pandemic as they relate to perceived health and housing conditions.

### Contextual policy documents

**Fig. 1:**
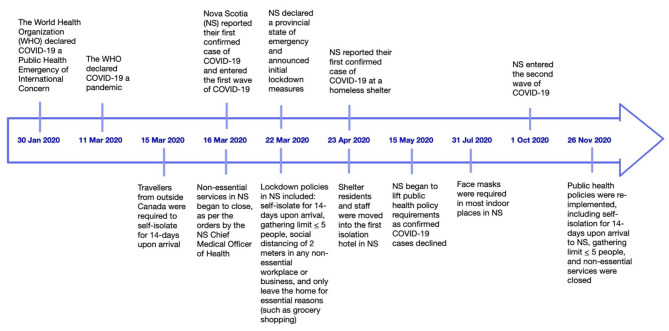
Pandemic timeline in Nova Scotia

Key policy documents were collected between April and December 2020 to capture evolving policy information. The documents provided context into the policies discussed with the participants during the interviews. We found 11 policy-related documents helpful in giving context to our interviews, including local, provincial, and national documents. A local document included a collective statement from the Executive Directors of four shelters in Halifax. Provincial documents included Nova Scotia’s Health Protection Act, the declaration of the Provincial State of Emergency, Provincial Health Authority policies and procedures on emergency preparedness (including the business continuity plan), and guidance on how to avoid infection (e.g., social distancing guidelines), masking guidelines, and symptoms and testing information. National documents included news releases introducing the Canada Emergency Response Benefit, updates on Reaching Home: Canada’s Homelessness Strategy during COVID-19, and the New Rapid Housing Initiative during COVID-19. Information about these documents, including title, source, type, topic area, date published, date accessed, audience, and a short description, was captured in a Microsoft Excel (version 2108) spreadsheet [28]. Using these documents and COVID-19 news releases from the Government of Nova Scotia [29], our team developed a pandemic timeline to situate ourselves within the research and capture background knowledge of the case context within which interview participants worked and lived (Fig. 1). The timeline reflects the rapid policy implementation following the first confirmed case of COVID-19, which ultimately led to a low case rate in Nova Scotia (144.06 cases per 100 000 population) compared to larger Canadian provinces, such as Ontario (1147.9 cases per 100 000 population) by the end of 2020 [30].

### Defining the case

**Fig. 2:**
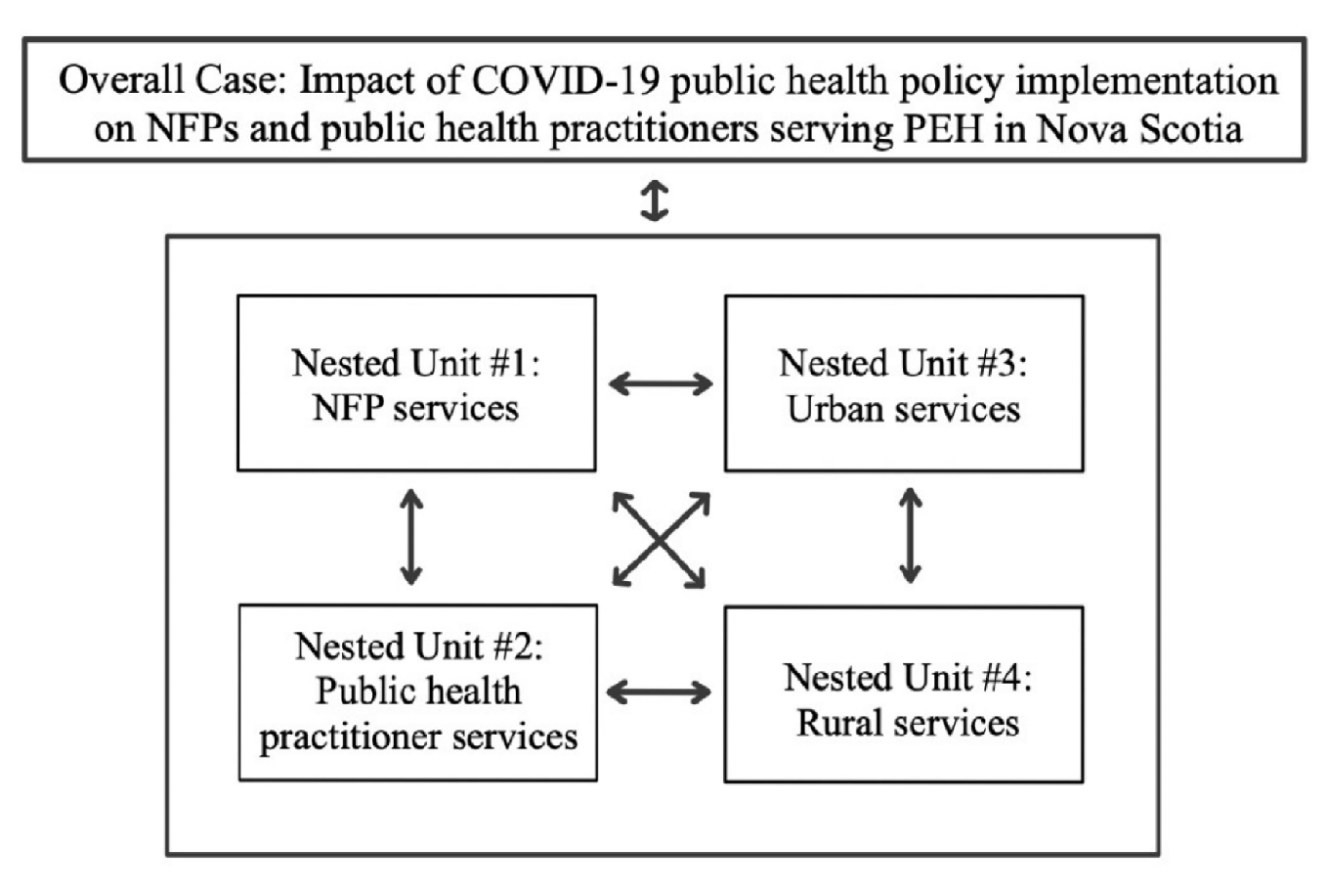
Diagram outlining the nested units compared to each other and the overall case

We used a single instrumental case study to understand a complex issue: the provision of services for PEH during the COVID-19 pandemic. Stake’s [31] definition of a case study emphasizes representing many perspectives without valuing one as better than the others, to capture the dynamic and context-specific information presented in the case. The case definition for this study was the impact of COVID-19 public health policy implementation on NFPs and public health practitioners serving PEH in Nova Scotia. A nested study approach examines multiple connected components within the case, known as nested units [32]. Nested units were used to compare findings within and between services and different places to understand the overall case. These four nested units were: 11) services that NFPs offer to PEH, 2) services that public health practitioners offer to PEH, 3) services in urban settings, and 4) services in rural settings. Within each nested unit were multiple organizations and service providers with various roles, responsibilities, and experiences working during a pandemic, as illustrated in Fig. 2. The case was bound by time (April 2020 – April 2021) and place (Nova Scotia, Canada).

### Data collection

Individual interviews were conducted with staff and leadership of NFPs and public health practitioners to elicit their experiences and perspectives about working with PEH. Technology limitations and distancing requirements posed challenges connecting with PEH during the pandemic; therefore, we did not include PEH as participants. We began recruitment by contacting our professional networks of public health practitioners and NFPs, who then referred us to potential participants and organizations. We also contacted NFPs using publicly available contact information on their websites. We emailed recruitment information describing our study to potential participants and offered optional virtual meetings to explain the study and answer any questions. Leadership within public health and NFPs helped to facilitate internal recruitment within their organizations, and interested service providers contacted us directly. Through these snowball and convenience sampling techniques, participants also met our eligibility criteria, including service providers serving PEH, 16 years or older, spoke English, and could verbally consent to be interviewed. We also ensured our sample included both staff and leadership of NFPs and public health practitioners based in urban and rural areas to reflect the nested units outlined in Fig. 2.

We obtained informed consent from all study participants following a consent discussion. Verbal consent was audio recorded and documented in writing by the team member facilitating the discussion. Interviews were conducted through a web-based platform and involved one or two qualitatively trained team members (A.D., B.C., or M.K.) and one participant at a time. Each interview lasted between 30 and 80 min, depending on how the interview progressed and participant preferences. Our research team consisted of women, except S.H. and B.C. Interviews took place between September 2020 and December 2020 during the first and second waves of the pandemic in Nova Scotia. As indicated in our timeline (Fig. 1), the first wave included the initial surge in cases at the beginning of 2020, and the second wave included the second surge in cases in the fall of 2020. A semi-structured interview guide was developed and pilot tested with two colleagues in the community with knowledge in this area. Participants could direct the conversation to the most relevant topics. Topics for discussion included the influence of COVID-19 outbreak control policies at a local, provincial, and systemic level, the impact of policies on participants, and their ability to influence policies (see supplementary material for interview guide). Interviews were audio recorded and transcribed verbatim by a team member or by using NVivo’s transcription service [33]. This service uses a powerful automated transcription technology to transcribe audio recordings. The research team reviewed the generated transcripts for accuracy, and all identifying information was removed. We did not conduct repeat interviews. Participants were offered a $50 gift card for their time and involvement in the study. This study was approved by IWK Health and St. Francis Xavier University Research Ethics Boards.

### Data analysis

NVivo (version 12.6.1) software was used for data management and retrieval [34]. We conducted a thematic analysis of the interview transcripts to identify patterns and generate themes [35, 36]. Following the phases of thematic analysis, A.D., B.C., and M.K. conducted the interviews and reviewed the transcription to enhance their familiarity with the data. These team members then ascribed codes to segments of the data to create a preliminary codebook. Through frequent meetings with D.H., S.H., and other team members, we compared and consolidated codes into themes [35]. At this stage of the analysis, we included the information from the contextual policy documents (based on the policies discussed during the interviews) to review and define themes. Using policies that influenced the services offered to PEH was important to contextualize participants’ experiences within the pandemic timeline and situate the themes within the overall case.

### Trustworthiness

Following Lincoln and Guba’s trustworthiness criteria [37], strategies to ensure dependability and confirmability included an audit trail of raw data, memos, and field notes. During data collection, we used field notes and memos to record unstructured observations, thoughts, and impressions during and after interviews to synthesize interpretations of the researcher-participant interaction [38]. Team debriefing after interviews and during analysis was essential to support credibility. Participants were invited to review their transcript (member check) to ensure it was accurate and confidential [31, 37]. We included rich and vivid descriptions of the case to fulfill transferability criteria so that readers can determine the applicability of these findings to their particular contexts [37].

## Results

The sample included 11 participant interviews (Table 1). Interviews were conducted with staff and leadership from rural and urban NFPs, as well as public health practitioners redeployed to support PEH in the broader provincial, rural, and urban contexts. The sample included a range of service providers with different roles and responsibilities during the pandemic, as outlined in our nested units (Fig. 2). There were similar numbers of participants between public health practitioners (*n* = 5) and NFP organizations (*n* = 6). Within the public health practitioners, there was an even distribution of participants covering provincial (*n* = 1), urban (*n* = 2), and rural (*n* = 2) jurisdictions. Within the NFPs, there were more urban-based organizations (*n* = 4) compared to rural-based organizations (*n* = 2). While we included more participants from NFPs in urban areas, our sampling reflected the available infrastructure to support PEH across Nova Scotia. Of the few participants we contacted who did not participate, their reasons for not participating included limited availability due to other professional responsibilities during the pandemic. We used information power to justify our final sample size [39]. As in all qualitative research, it was difficult to know whether our sample captured every instance of COVID-19 policies impacting services supporting PEH. Instead, having adequate information power meant that participants adequately described the services in urban and rural areas, including the perspectives of public health practitioners and NFPs. The 11 policy documents were then used to contextualize policies discussed during the interviews.

**Table 1:** Participant and organization descriptions

Type of organization	Description of organization	Number of participants
Public Health Practitioners	Public health practitioners from the provincial health authority were assigned to support the organizations serving PEH during the pandemic. Responsibilities included assisting context-specific public health policy implementation while continuing essential care for PEH.	*n* = 5
NFPs		
Adult emergency homeless shelters	Temporary shelters for those who identify as men (including gender-diverse individuals) and those who identify as women (including gender-diverse individuals) experiencing homelessness. Located in urban Nova Scotia, some services include meals, phone and laundry access, access to personal care items (such as socks and toiletries), and housing worker assistance. Onsite health services are also offered through community-based outreach programs.	*n* = 3
Youth homeless shelter	Temporary shelter for youth (16–24 years old) experiencing homelessness. Located in rural Nova Scotia, some services include meals, programs and workshops, phone and laundry access, referrals to other services, and outreach health services.	*n* = 1
Shelter and transition house for women and their children	Temporary shelter and transition house for women and their children who are experiencing violence and abuse. Located in rural Nova Scotia, some services include meals, crisis intervention, supportive counselling, advocacy work, referrals, and outreach services.	*n* = 1
Primary health care services	Interdisciplinary health care team offering primary health care services to PEH. Located in urban Nova Scotia, services are delivered through community-based, outreach, and onsite programs in collaboration with shelters, food services, and other organizations.	*n* = 1

Three themes were generated from the interview data analysis: *a perfect storm*, *some assembly required*, and *behind the mask*. *A perfect storm* illustrates the complexity of issues among PEH, and the barriers service providers faced to addressing homelessness and implementing policies during the pandemic. *Some assembly required* describes how service providers navigated these barriers and translated written policy into action. *Behind the mask* details the personal burden on service providers by the pressures of working amid the pandemic. Throughout the results, we have referenced policy documents that helped form our understanding of the theme.

### A perfect storm

The first theme illustrates a combination of factors that created an effect greater than the sum of its parts; these factors included the challenges service providers faced in implementing COVID-19 outbreak control policies. Participants described the intersectoral services needed to address the complex needs of PEH, including gender-based violence support, food services, harm reduction interventions, and mental health treatment.

During the initial state of emergency, the lockdown period included strict policies outlined in our timeline (Fig. 1). Non-essential services were closed per the orders of the Nova Scotia Chief Medical Officer of Health under the Health Protection Act [2]. Workplaces providing essential services, such as health, food, and transportation, implemented distancing and gathering limits to minimize contact between and among clients and staff. There was often confusion for NFPs around what was considered an essential service and how to continue supporting PEH in the community. Public health practitioners continued essential and feasible services through a business continuity plan, but most roles shifted to the COVID-19 response as other public health initiatives were halted. As participants quickly moved into pandemic response roles, it is important to note homeless shelters were exempt from physical distancing requirements and gathering limits [2]. However, there were still expectations for shelter workers and clients to maintain distance to the best of their ability. Due to a lack of pre-existing evidence and guidelines to prevent disease transmission within shelters, there was a lot of uncertainty early in the pandemic:

*“It was interesting to try and figure out what was best practice for shelters*,* because there’s not a lot of material out there. And like when you google “homelessness and pandemic*,*” I think a lot of that came out of COVID.”* (Participant #11).

As service providers were figuring out how to implement new public health policies to keep people safe, they also supported people experiencing gender-based violence, food insecurity, substance issues, and mental health conditions, adding another layer of complexity to service delivery. Participants described difficulty enforcing physical distancing and self-isolation policies for many PEH:

*“The part that was extremely difficult was around the complexity of supports that are required for this population [PEH who use substances] and putting them in self-isolation in a hotel*,* like not able to get access to the substances that they’re addicted to. How do we provide harm reduction support? How do we provide mental health supports?”* (Participant #1).

NFPs were excluded from public discourse early in the pandemic. For example, the phrase “stay the blazes home” [40], popularized by the Premier of Nova Scotia at the beginning of 2020 during a press conference, highlighted the mismatch between the language used to promote compliance with public health mitigation strategies and the realities of the NFP sector:

*“I think what really hit home was the “stay the blazes home” language that we were hearing. And it really*,* really shone a light on the ways that our population was disproportionately affected by the pandemic because people have no home. So*,* of course*,* this message of “stay the blazes home” and “stay six feet apart” was completely falling to the wayside for us because there was no ability to social distance.”* (Participant #10).

Systemic barriers such as overcrowding, inadequate funding and resources, lack of political motivation to implement new policies, and the underlying assumption that people had access to safe and secure living spaces led to difficulty in implementing public health measures. Participants noted that most housing options provided for PEH offered short-term solutions, such as shelters. While homeless shelters have always been vital partners for addressing acute and emergent needs, the paucity of resources available to support PEH posed challenges for service providers. While housing is a social determinant of health and directly related to health outcomes, the infrastructure to support PEH is focused on emergent short-term needs.

*“Housing is health and shelters are not a solution to homelessness. And they’re often operating with the bare minimum in terms of resources and supplies. And when you throw something like a pandemic into the mix*,* you realize how fragile our system is and how the real solution would just be to have affordable*,* supportive housing.”* (Participant #11).

The pandemic response also magnified rural housing disparities observed by service providers. NFPs in rural areas often receive a disproportionate amount of funding for services compared to urban areas. There were also transportation and technology limitations impacting services. For example, the main public transit system is in Halifax, and the rural areas rely on community-based transportation companies. A participant described the challenges coordinating transportation for PEH, especially at the beginning of the pandemic when people were urged to stay home, and public transit was significantly reduced or non-existent in some rural areas:

*“I always see the experience in [urban city] as very different. I know some colleagues who work in other [rural] areas of the province and transportation was a much bigger issue. They don’t have the same consistent level of transportation services for people who did need to get to a testing center from a shelter or just [in] general.”* (Participant #2).

At the beginning of the pandemic, libraries, parks, public washrooms, and other community-based resources were not considered essential services [2]. These important services were either closed or moved online, straining the already limited resources available for PEH. A rural-based participant recalled droughts in their area, and the challenges supporting PEH when water access was limited:

*“We have experienced an extensive drought at this end of the province this summer. And although it is a regularly occurring thing where folks here who are on wells*,* their wells go dry*,* it typically doesn’t last months and months. This summer*,* it lasted months and months. And so we worked with our municipal folks to get water distribution set up among a pandemic… If you’re homeless and you have nowhere to be able to do those kinds of things with public access to showers and places to wash closed*,* it adds again another layer of chaos.”* (Participant #4).

The complex services needed to support PEH during the pandemic illustrated the pre-existing inequities for PEH perceived by service providers. Without established best practices and context-specific action plans, COVID-19 policies outlined by governmental agencies would not work for services designed to support PEH. In the second theme, we describe how participants interpreted, conceptualized, and implemented COVID-19 public health outbreak control policies.

### Some assembly required

Although there were strategies to prevent the spread of COVID-19 (such as physical distancing, self-isolating, and working from home), many of these policies were difficult to implement in shelter settings. The NFP sector needed to adapt to the evolving nature of the pandemic using an intersectoral approach through governmental and community-based wraparound services. Wraparound support from a team of health and social sector workers aimed to address various needs of PEH, such as mental health, harm reduction, and housing. The theme *some assembly required* describes the unique ways service providers adapted and developed new infection control strategies to prevent the spread of COVID-19. As part of these wraparound services, homeless shelters were altered, temporary pop-up shelters and isolation hotels for PEH were created, phone help lines were implemented, and transportation was organized.

As widespread messaging and language for COVID-19 control policies largely omitted guidance for PEH, public health practitioners and NFPs said they had to rapidly find solutions. To physically distance, shelter workers reduced capacity and renovated bed areas. The following document excerpt was included to contextualize participant interviews among the current policies that were being implemented:

*“Even before the pandemic*,* beds were full in our non-profit sheltering and housing organizations. We often had to turn as many people away as we were able to welcome in. When the COVID-19 hit*,* we adapted our protocols and physical spaces overnight. We reduced the number of beds in order to maintain physical distancing and increase safety for all who stay and work in shelters. This has come at a cost… Across HRM [Halifax Regional Municipality]*,* this translates to a shortage of 17*,*520 beds for people over the course of a year*,* where people have no safe place to be during a global pandemic.”* (Local collective statement document prepared by Executive Directors of four shelters within the Halifax Regional Municipality, titled *“*No Time to Waste: Solutions to the housing emergency are paramount in the fight against COVID-19 and beyond*”*).

The following participant reinforces the “costs” of reducing shelter capacity described in the document excerpt above, including living on the street and in other potentially unsafe environments:

*“It’s a really big concern to the point that*,* you know*,* they’ve started documenting how many people they were turning away and more of what I think is probably the first time in [urban center] history that we have somewhat of a tent city starting to emerge. People living on the street because there just aren’t enough shelter spaces for them to be accommodated and still maintain the physical distancing.”* (Participant #1).

As a result of decreased capacity, three temporary pop-up shelters were created by re-purposing schools, hotels, and community centres in [urban center referenced by Participant #1] that had been closed to comply with public health guidelines. However, finding qualified and experienced staff to work in these new pop-up shelters was difficult. Shelter organizations had staffing issues before the pandemic; this was exacerbated by stressful working environments, fears of contracting the virus and spreading it to others, and staff unavailability due to the 14-day isolation requirement for anyone who had travelled, been exposed to, or contracted COVID-19.

*“We have not enough staff because people are either afraid to come to work or they’re getting tests themselves*,* so they’re not allowed to come to work. And we’ve lost tons of staff throughout this process*,* of course*,* because there’s people that were not willing to come work in these high-risk environments anymore.”* (Participant #10).

Comparatively, participants from a youth shelter and transition house in rural Nova Scotia experienced a decreased demand for their services at the beginning of the pandemic. The decreased demand may have been attributed to people reconnecting with estranged family members, fear of contracting the virus from accessing services, and uncertainty if services were still being offered. The following participant described the concern for clients being unable to access services during the pandemic, especially for women and children experiencing gender-based violence in the home:

*“The communication around [lock]-down I think meant that people stayed at home and didn’t access services. So we certainly did see that in our community and we saw a decrease in the number of folks accessing our transition houses and the number of calls that came in. People didn’t anticipate anything being open or that there were services. So those were areas of concern for our community.”* (Participant #4).

Public health practitioners worked closely with NFPs serving PEH to support isolation, point-of-care testing in shelters, and transportation between shelters and testing centres. As policy guidance evolved, a dedicated phone line was an important tool developed in response to the urgent need for appropriate housing among PEH. Rather than calling a province-wide helpline for COVID-19 guidance and information, public health practitioners partnered with NFPs to create the housing isolation phone line. The following participant described a positive response that NFPs had to calling the housing isolation phone line directly for advice and assistance on implementing policies:

*“One of the significant things I would say public health has done is they created a secondary line*,* like the HIP [housing isolation phone] line for us to call. So we don’t call 811. We actually have a dedicated line that’s available to us seven days a week from 8 a.m. to 6:30 p.m. And we can call there and they specifically work with our guests.”* (Participant #10).

When a positive case was first identified in a shelter, the provincial government funded and moved shelter staff and clients into a hotel sitting vacant due to COVID-19-related closures to decrease the further spread and promote isolation efforts. Moving entire shelters into hotels during outbreaks created additional considerations for food distribution, mental health support, and people who use substances. For example, participants described the challenges supporting people who used substances and were unable to access their regular supply in the hotel setting:

*“At one point we did have a positive [case] in the shelters and so we moved all of those two shelters into hotel rooms*,* separated them*,* and then they had to isolate. However*,* these guys are really heavy users and A) would go into severe withdrawal*,* but also just wouldn’t be able to adhere to isolation because of their cravings. And so my colleague and I […] just had to get pretty ballsy and decided to do a harm reduction approach.”* (Participant #8).

An example of a harm reduction initiative implemented during the pandemic was a managed alcohol program coordinated by NFPs and funded by the provincial government. The program involved health care providers in Halifax prescribing safe and stable doses of alcohol to people with severe alcohol use disorders experiencing homelessness to reduce harms associated with uncontrolled alcohol use, non-beverage alcohol use, and withdrawal:

*“Nova Scotia has seen its first managed alcohol program as a result of COVID responses*,* which is incredible. And those are looking to be pilot [programs] that we’re trying to renew or extend. So like that’s a huge*,* huge change. That is a result of people taking more*,* I think*,* doing more things than they would have done previously because COVID forced us to work differently. And it also really shone a light on the very specific needs*,* like I said*,* of this*,* of this particular population and ways in which they’re supported or not supported.”* (Participant #10).

Participants also discussed the process of forming interdisciplinary partnerships to address service gaps and facilitate transportation, testing, and isolation measures:

*“Initially*,* my role was focused in on helping folks who required accommodation for isolation purposes because they could potentially have COVID-19. So we had to work with community around trying to source out places*,* hotels in particular*,* where it would be willing to house folks who might require accommodation*,* support for isolation*,* as well as transportation and food while those folks were being housed.”* (Participant #4).

Public health practitioners worked with community organizations to open comfort stations around the province and mitigate the impact of closing public spaces. When PEH could not access shelters due to outbreaks, lockdown measures, or limited beds, comfort stations offered a temporary place to go:

*“They got open what they call comfort stations with places that people go to access washrooms that included showers*,* a laundry facility to wash their clothes*,* and places to come and have a cup of tea or coffee or something like that to warm up.”* (Participant #1).

Especially in rural settings, the community response filled in the service gaps caused by transportation limitations and closing public spaces. One participant commented on the community response needed in rural areas to address food insecurity among PEH, which often included community member volunteers and faith-based organizations:

*“We saw lots of community responses around food delivery and grocery store delivery*,* mobilizing of different groups to help fill some of those gaps.”* (Participant #4).

Adaptation and implementation of new programs and services aimed at supporting PEH during the pandemic would not have been possible without collaboration between participants and representatives from many different services and sectors, including community volunteers, hospitality, and transportation, as well as government representatives engaged in the administration of federal and provincial funding initiatives. Participants worked tirelessly to implement policies and serve PEH, and in the next theme, we will describe how the pandemic professionally and personally impacted them.

### Behind the mask

As service providers continued to support their clients by following and implementing infection control policies, they were themselves working and living through the pandemic. Participants discussed with us the challenge of providing services to others while at the same time managing their own physical and mental health. This is summarized in our third theme, *behind the mask*, where we explore participants’ reflections on the personal and professional impacts of serving PEH during the pandemic.

Participants were fearful of bringing the virus into the workplace when working in a high-risk shelter setting. Apart from limitations created by travel restrictions, gathering limits, masking, and distancing policies, participants were sometimes unable to work due to exposure or experiencing any known signs and symptoms of COVID-19. One participant described feeling guilty about being unable to provide vital services:

*“You know*,* most people come into work*,* come hell or high water. And so there was a lot of support that needed to happen to our colleagues because of the guilt that they felt because they had a sore throat. They couldn’t do the work that they wanted to do. And I think people take their job seriously here. And we all knew at the time how important our work and presence was because there was so little of it.”* (Participant #8).

A lack of extracurricular activities, social interactions, vacations, and coping mechanisms to deal with the heightened stress had profound personal implications. In addition to staffing shortages and increased workloads and responsibilities, many participants described feelings of burnout and its associated mental health implications:

*“I noticed there was lots of times where I felt like I had way less patience. I was more snappy. I definitely noticed a decrease in my ability or my want to fix situations. Sometimes I would just throw up my hands and be like*,* well*,* they’re just going to have to sleep outside tonight. And like*,* that has never been me. And so then having that introspective moment of feeling guilty about how I’ve become more desensitized and dulled to lots of deaths*,* and we had lots of deaths. So then the interesting point of feeling like not affected by the deaths like I used to*,* um*,* and then feeling guilty about that and so lots of just mental health stuff.”* (Participant #8).

Ultimately, the pandemic changed how we work with others. Working from home posed technological barriers to connecting with PEH, and most participants continued to work in-person. As distancing and isolation measures limited movement within the community, service providers had more consistent contact with PEH. While everyone shared a similar risk of contracting COVID-19, constant contact between staff and clients may have shifted attitudes towards PEH and helped build positive relationships between staff and clients:

*“Something is affecting us as much as it’s affecting the guests. Even though we deal with homelessness every day and it takes quite a burden*,* at the end of the day*,* all of the shelter employees are able to leave and go to their home. But we were sort of noticing like*,* wow*,* now the risk is the same for all of us. We’re experiencing something to the same degree our guests are.”* (Participant #11).

Moreover, implementing COVID-19 outbreak control measures within NFPs may have negatively impacted relationships between staff and clients. Outbreak control policies, such as stopping drop-in services, adding physical barriers (such as plexiglass), and restricting in-person activities, changed some service delivery models:

*“So it [COVID-19 policies] kind of took that personable aspect out of it and kind of made it seem like we weren’t necessarily as accessible and supportive as we used to be.”* (Participant #7).

Along with staff and client relationship changes, NFPs and public health practitioners formed collaborative, intersectoral partnerships which invigorated the pandemic response and fostered improved knowledge and resource sharing between stakeholder groups:

*“We have never felt like the health authority or public health has respected our work or really appreciated our work. And often they ask us to do really inappropriate things. And we have always felt that they could get out into the community more than just sitting using phones. And our clients don’t have phones. So they kind of saw how we did that and they were very involved. And then from that came a really beautiful relationship between public health and us*,* where I felt like their respect for us really improved. And they actually started asking for our opinions and we started to work very collaboratively. And they changed their practice a lot. And I really give them kudos because it’s not easy in a big institution*,* but they’ve really*,* really stepped up. And yeah*,* I feel like that’s only going to be helpful for everyone going forward.”* (Participant #8).

Difficulties navigating a novel pandemic coupled with stressful working conditions led to fear, uncertainty, and changes in working conditions. It is crucial to capture the unique challenges faced by providers serving PEH to inform future emergency preparedness measures, as discussed in the following section.

## Discussion

While public health policies have been instrumental in the COVID-19 response, a lack of pre-existing emergency preparedness and response plans and the chronically strained NFP sector led to COVID-19 policy implementation gaps [21, 41, 42]. Through this qualitative case study, we highlighted what happened in the non-profit sector serving PEH during COVID-19, and the strategies used to implement and promote adherence to public health outbreak control policies. These findings contribute to the growing body of research demonstrating housing as an essential social determinant of health and front-line defence against disease.

Different iterations of outbreak policies were introduced to match evolving scientific knowledge about the virus as the pandemic progressed. While NFPs and designated public health practitioners were instrumental in coordinating the COVID-19 response in the NFP sector, they needed more guidance, resources, and infrastructure to address housing issues. As reflected in our timeline (Fig. 1), many policies were rapidly implemented across local, provincial, and national levels. Service providers working with PEH had difficulties fitting these policies to their specific contexts and clients’ needs. As the provincial government implemented temporary pop-up shelters, service providers working with PEH anticipated challenges in finding trained and qualified staff to work at these sites. As a result, staff from NFPs in the community took on the responsibility of supporting these shelters, in addition to the demands of their existing roles. The predicted staffing issues for pop-up shelters highlighted the importance of seeking input from service providers in the NFP sector to inform and implement feasible public health measures. These findings are consistent with two qualitative studies conducted in Canada, which also found a need for more feedback from front-line service providers on outbreak policies at the beginning of the pandemic [43, 44].

The example of pop-up shelters also illustrated that service providers working with PEH in the community are often the only reliable and accessible resource for PEH, creating additional responsibilities and workloads. This reality meant that NFP staff experienced high levels of job-related stress and burnout. Compounded by the stressors of the COVID-19 pandemic, there were personal impacts on service providers using limited resources to manage complex clients. Even before the pandemic, a 2016 study conducted in Edmonton, Canada, used survey data from 234 providers serving PEH and found that 21.9% of participants reported high levels of secondary traumatic stress, and 23.2% reported high levels of burnout [45]. Emergency preparedness plans must prioritize clients’ and service providers’ physical and mental health. Governmental and organizational policymakers must work together to ensure adequate working conditions and compensation for community-based service providers to retain staff and prevent shortages [46]. While recovering from the COVID-19 pandemic and planning for future emergencies, staff should be offered mental health and wellness resources and adequate training opportunities [46]. Overall, these service providers need to be acknowledged as essential workers in any emergency and given context-specific guidance.

It is also important to note the differences between urban and rural services. Service providers in rural settings have faced additional challenges amid the COVID-19 pandemic. Barriers to serving clients in these areas included lack of transportation and internet access. The pandemic exacerbated these pre-existing issues, as travel restrictions, physical distancing, and the transition to online services likely excluded people from accessing rural services. Furthermore, without access to accurate rural homelessness statistics, rural housing initiatives may not be able to substantiate a need for services in funding applications [18, 47, 48]. As rural homelessness is becoming recognized as a substantial issue, researchers and advocates recommend research networks focusing on rural homelessness across Canada [6]. Capturing rural service delivery experiences is important to inform subsequent programs and policies. Triangulating the experiences of public health practitioners and staff and leadership of NFPs in urban and rural areas, as well as contextualizing these findings within the policy documents, is a considerable strength of this research. Intersectoral collaboration was instrumental in implementing outbreak policies, and if these partnerships are left in place after the pandemic, can benefit other health and social initiatives [49].

Based on the findings from our study, service providers recognized the COVID-19 pandemic disproportionately impacted people contending with intersecting challenges, including homelessness and co-occurring substance use and mental health issues. A nationally coordinated Point-in-Time count including 19 536 people’s experiences of homelessness across 61 Canadian communities in 2018 found that 25.1% of respondents recorded addiction or substance use as their reason for most recent housing loss [50]. The proportion of people who reported addiction or substance use was 19% among those who experienced up to two months of homelessness, and 28.2% among those who experienced over six months of homelessness in the previous year [50]. The complex relationship between homelessness and substance use necessitated harm reduction strategies (reducing adverse consequences of substance use when decreasing or eliminating use is not feasible) during lockdown and isolation periods. A study using data from isolation hotel shelter residents following a COVID-19 outbreak in May of 2021 in the congregate shelter system in Halifax found that the temporary safe supply program administering pharmaceutical-grade medications was associated with low rates of adverse events (including overdose, intoxication, diversion, selling, or sharing of drugs or alcohol) and high rates of completing the 14-day isolation period [51]. However, a government and public health decision to withdraw funding for isolation hotels and safe supply medications after the isolation period prevented long-term evaluations, including whether the supportive housing model led to permanent housing and improved chronic health conditions [51]. Service providers discussed the challenges of addressing co-occurring substance use and mental health disorders among clients during the pandemic, indicating an important area for organizations to facilitate further education and training.

This study has potential limitations. Interviews were conducted over web-based platforms, which limited non-verbal cues and communication, especially in rural areas where participants’ Wi-Fi did not support video. Interviews were completed early in the pandemic and only reflect the events experienced by service providers in Nova Scotia during this time and before the availability of vaccines. Finally, these findings do not represent the voices of PEH due to technology limitations and physical distancing requirements.

## Conclusion

It is important for policymakers, funding agencies, and front-line service providers to understand the critical factors that support and inhibit the implementation of COVID-19 public health outbreak control policies on services offered to PEH. As policies were made to prevent and control COVID-19 outbreaks, front-line service providers were instrumental in adapting policies to their setting and clients’ needs. Along with people with lived experiences, it is essential to include service providers working with PEH in the policy decision-making process. Intersectoral collaboration was identified as an integral component of policy implementation, including designated public health practitioners supporting the NFP sector. The negative personal impacts on essential service providers working with PEH reinforce the need for supportive and healthy work environments. Participants in our study told us how they had to act quickly and creatively to mitigate the unintentional consequences of COVID-19 prevention and control policies, including renovated shelters, designated pop-up shelters, comfort stations, coordinated testing, managed alcohol programs, and phone helplines for shelter staff. While these efforts were needed during the COVID-19 pandemic, the housing crisis has been a longstanding issue across Canada. To better support PEH during public health emergencies and mitigate the impacts on housing services, it is crucial to recognize housing as a social determinant of health and strengthen infrastructure around homelessness responses through funding initiatives and collaborative, intersectoral services. This study reflects the need to focus on long-term, safe, and stable housing, low-barrier outreach services, as well as supportive public and social policy.

While the pandemic increased public awareness of local, provincial, national, and international housing needs, the momentum and political will to address the systemic causes of homelessness must persist in the post-COVID-19 era. Acknowledging and embracing the distinct rural challenges (including limited resources for PEH) and the context of homelessness in different settings is essential to form and implement meaningful policies. As public health policies exacerbated pre-existing issues in the NFP sector, the pandemic highlighted a need for action at all levels within a complex system to ensure widespread, sustainable change.

## Electronic supplementary material

Below is the link to the electronic supplementary material.

Supplementary Material 1

## Data Availability

The data generated and analyzed during the current study are not publicly available to ensure confidentiality of study participants but are available from the corresponding author on reasonable request.

## Abbreviations

COVID-19: Coronavirus disease of 2019
NFP: Not-for-profit organizations
PEH: People experiencing homelessness
